# Whole-Exome Sequencing Could Distinguish Primary Pulmonary Squamous Cell Carcinoma From Lung Metastases in Individuals With Cervical Squamous Cell Carcinoma

**DOI:** 10.3389/pore.2022.1610325

**Published:** 2022-05-11

**Authors:** Lihong Li, Qianqian Song, Dandan Cao, Yuchen Jiao, Guangwen Yuan, Yan Song

**Affiliations:** ^1^ Department of Pathology, National Cancer Center/National Clinical Research Center for Cancer/Cancer Hospital, Chinese Academy of Medical Sciences and Peking Union Medical College, Beijing, China; ^2^ State Key Lab of Molecular Oncology, Laboratory of Cell and Molecular Biology, National Cancer Center/National Clinical Research Center for Cancer/Cancer Hospital, Chinese Academy of Medical Sciences and Peking Union Medical College, Beijing, China; ^3^ Genetron Health (Beijing) Co. Ltd., Beijing, China; ^4^ Department of Gynecology Oncology, National Cancer Center/National Clinical Research Center for Cancer/Cancer Hospital, Chinese Academy of Medvdical Sciences and Peking Union Medical College, Beijing, China

**Keywords:** metastasis, cervix, squamous cell carcinoma, sequence, lung

## Abstract

**Aims:** Metastatic cervical carcinoma is hard to cure using traditional treatment and new therapeutic approaches are needed. However, the process of clonal evolution and the molecular alterations that contribute to tumor progression from primary to metastatic carcinoma remain unclear. It is currently difficult to distinguish between the primary pulmonary squamous cell carcinoma (PPSCC) and metastatic cervical squamous cell carcinoma (CSCC).

**Methods:** Paired primary CSCC and lung/lymph nodes metastatic lesions from eight patients were analyzed by whole-exome sequencing (WES). WES data of matched specimens and normal samples were aligned to the human reference genome and analyzed to identify somatic mutations in primary and metastatic lesions.

**Results:** A total of 1,254 somatic variants were identified. All the primary lesions and metastatic lesions shared mutations, the percentage of shared mutations between primary lesions and corresponding metastatic lesions varied significantly, ranging from 6% to 70%. In other words, all the metastatic lesions are clonally related to primary lesions, confirming WES could prove they are metastatic from the cervix but not PPSCC. We tried to apply a gene panel to help distinguish PPSCC and metastatic CSCC but failed because the mutations were widely distributed in CSCC. Interestingly, lymph nodes metastasis (LNM) harbored fewer cancer driver mutations than primary CSCC specimens with a significant difference. Besides this, there was no significant difference in somatic mutations and copy number variation (CNV) between primary and metastatic CSCC.

**Conclusion:** Our data demonstrate that WES is an additional helpful tool in distinguishing PPSCC and metastatic CSCC, especially for certain difficult cases.

## Introduction

Cervical carcinoma is the third most common gynecologic cancer and the fourth leading cause of cancer death worldwide [1]. Human papillomavirus is central to the development of cervical neoplasia and can be detected in more than 90 percent of cervical cancers [2]. The most common histologic types of cervical cancer are CSCC. The pelvic nodes and lung are the most frequent metastatic sites for CSCC [3]. For patients with cervical cancer, 4.16–7.7% of patients have lung metastasis [4].

One problem that exists in our routine work is that it is hard to distinguish between metastatic CSCC and primary lesion. If a solitary lung squamous cell carcinoma is found in a patient with CSCC history, the lesion could be either metastasis CSCC or a PPSCC. The differential diagnosis has a big difference in treatment and prognosis. Lung metastasis (LM) is formed through hematogenous metastasis of CSCC and originates from primary CSCC clones. At the same time, PPSCC is generally believed to originate from independent clones from primary CSCC. The treatment is also different for the two diseases; further chemotherapy is needed for LM, while PPSCC cases require surgery. Thus, distinguishing PPSCC from LM is important since the diagnosis can affect subsequent treatment strategies and survival evaluations. In clinical practice, we compare the morphology similarity of lung tumor and CSCC; if they are similar, we tend to believe they are metastatic lesions, and vice versa. However, the morphology is unreliable because the CSCC and PPSCC could be pretty similar. And the p16 positive expression can’t help differential diagnosis because the p16 staining could also be strongly positive in PPSCC cases [5–7]. HPV genotyping [7, 8] could only discriminate about half of these controversial cases because PPSCC could also have HPV infection status. Thus, in this study, we tried to apply WES and analyze if they are clonally related to help distinguish LM and PPSCC.

Metastatic cervical carcinoma is hard to cure using traditional treatment, so new therapeutic approaches are needed. Previous studies demonstrated the function of *PTEN*, *PIK3CA*, *TP53*, and *KRAS* in the pathogenesis of CSCC [9–12]. The genomic landscape was well established in 2017 by The Cancer Genome Atlas (TCGA) Research Network, which increased our understanding of CSCC [13]. They observed some novel mutations in CSCC, like *SHKBP1*, *CASP8*, *HLA-A*, and *TGFBR2*. Despite this advancement, a comprehensive molecular landscape depicting the genome alterations during the tumor progression from primary CSCC to metastatic diseases remains elusive. The molecular pathogenesis of metastatic CSCC and how primary CSCC progresses to metastatic CSCC remain largely unclear as genome-wide molecular genetic analysis of metastatic CSCC has not been reported. Revealing this could help us acquire a deep understanding of the genomic process of metastasis and may help us develop new targets on preventing metastasis and improving survival. This study performed WES in surgical specimens of eight pairs of primary and metastatic CSCC. And we analyzed somatic mutations including cancer driver mutations and CNV between primary and metastatic CSCC.

## Materials and Methods

### Tumor Specimen

All eight cases were selected from Cancer Hospital, Chinese Academy of Medical Sciences after approval by the Institutional Review Board. The sequence cohort was selected based on the availability of paired tumor primary and metastatic tissues and matched normal tissue controls. All cases received the total hysterectomy surgery. Two experienced pathologists confirmed the diagnosis (L. L. and Y. S.). CSCC1-CSCC3 had paired primary CSCCs and LM specimens, and CSCC4-CSCC8 had paired primary pelvic LNM samples. CSCC1 had two LM specimens, which were LM1 and LM2. The interval from LM1 to LM2 was 12 months for case 1. Finally, 25 FFPE samples, including the paired normal control samples from eight patients with CSCCs (CSCC1- CSCC8), were submitted to WES.

### Somatic Mutations in Paired Primary and Metastatic CSCC

Tumor and normal DNA were extracted from tissue shavings of formalin-fixed and paraffin-embedded specimens using QIAamp DNA FFPE Tissue Kit (Qiagen, Valencia, CA, United States) according to the manufacturer’s instructions. DNA was quantified by Qubit (Life Technologies).

According to the manufacturer’s instructions, WES was performed on the genomic DNA obtained from tumor tissues and matched normal tissue using the Agilent Sure Select Human All Exome V5 Kit (Agilent Technologies, Santa Clara, CA, United States). Paired-end sequencing, resulting in 150 bases from each end of the fragments, was performed using the HiSeq X Ten. The paired-end sequencing raw data (FASTQ format) were aligned to the human reference genome (hg19) using Burrows-Wheeler aligner software (BWA, v0.7.15). The Genome Analysis Toolkit (GATK, v3.6), Picard (http://picard.sourceforge.net; v2.7.1), and Samtools (v1.3.1) were used for the basic processing and management of marking duplicates, including local realignments and score recalibration. Somatic mutations were detected by comparing tumor and matched normal sequencing data using MuTect1 and Strelka for single-nucleotide variants and InDels. Somatic mutations in the test samples were defined according to the following criteria: 1) the mutation was identified in four or more distinct reads and the mutation depth was greater than 15 reads, 2) the mutation was not present in any of the reads in the matched control sample or the other control samples analyzed in this study, and 3) the mutation was uncommon in dbSNPs (frequency < 1%). Only non-synonymous were reported. Putative driver mutations were determined based on previously listed cancer driver genes [14]. All candidate somatic mutations were validated by visual inspection using the Integrated Genome Viewer (IGV) [15].

Moreover, ANNOVAR was used for the functional annotation of each variant in the coding regions. PolyPhen-2 was utilized to predict the impact of an amino acid substitution on protein function and structure.

### Detecting Copy Number Variation and Clonal Composition Using WES

Binned copy number and segmentation data of the tumor genome, compared to the matched normal tissue, were computed using the copy number calling pipeline of alignment data in the CNVkit package [16]. The visualization of log-ratio segments was done using matplotlib plotting library.

### Phylogenetic Analysis

We used the “discrete‐characters Wagner parsimony” method in phylogeny inference package (phylip) version 3.698 [17] to generate phylogenetic trees. All phylogenetic trees were rooted using the control tissue DNA, which represents the ancestral state. Cladograms were plotted with the number of mutations per branch at each end. The trees were drawn using the DrawTree tool under the phylip package.

### Patient and Public Involvement Statement

Patients were involved in the design and conduct of our research.

### Statistical Analysis

Statistical analysis was done with Statistical Package for the Social Sciences (SPSS 22.0). In all experiments, comparisons between two groups were based on the Wilcoxon test. Mann-Whitney U was utilized to test for differences among groups. *p*-values of <0.05 were considered statistically significant.

## Results

### Clinicopathological Characteristics

The clinicopathological information of the eight CSCC patients was summarized in Table 1. The median age of these patients was 48 years old. The histopathological features in a representative case are illustrated in Supplementary Figure S1. Four patients had poorly differentiated carcinomas, three had moderately differentiated carcinomas, and one had well-differentiated carcinoma. International Federation of Gynecology and Obstetrics (FIGO) stage is IB1 (1/8), IB2 (5/8), and IIA (2/8), respectively. The average months from the primary tumor to the LM tumor was 37 months. All patients received neoadjuvant therapy after the surgery.

**TABLE 1:** Clinicopathological characteristics of eight patients.

Case ID	Gender	Age of first-time diagnosis (yrs.)	Smoking status	FIGO stage	Histology	Differentiation	Present with distance metastases at first-time diagnosis
CSCC1	Female	45–50	No	IIa2	SCC	Poor	Y
CSCC2	Female	35–40	No	Ib2	SCC	Moderate	Y
CSCC3	Female	50–55	No	Ib2	SCC	Poor	Y
CSCC4	Female	45–50	No	Ib2	SCC	Moderate	N
CSCC5	Female	45–50	No	Ib2	SCC	Poor	N
CSCC6	Female	45–50	No	Ib2	SCC	Well	N
CSCC7	Female	50–55	No	IIa1	SCC	Poor	N
CSCC8	Female	45–50	No	Ib1	SCC	Moderate	N

### The Comparison Between Primary and Metastatic CSCC Carcinoma

As shown in Supplementary Table S1, the average target depth for the analysis was 182.3×, 98.9% of which was covered by more than ten reads. One thousand two hundred fifty-four non-synonymous somatic mutations were identified. The complete list of somatic variants was presented in Supplementary Table S2. From these, 91 canonical cancer-driver mutations were detected (Supplementary Table S3).

**FIGURE 1:**
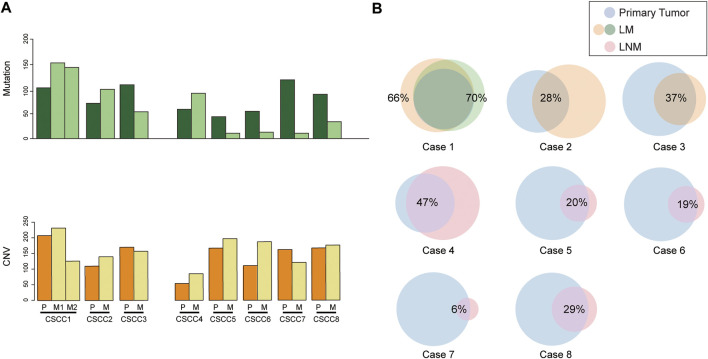
Comparison of somatic mutations in paired primary CSCC and metastatic CSCC. **(A)** mutation burden of eight CSCC pairs. **(B)** Venn diagrams depicting the overlap in somatic mutations in paired primary CSCC and metastatic CSCC. CSCC: cervical squamous cell carcinoma; P: primary lesions; M: metastatic lesions (lung metastasis in case 1–3; lymph nodes metastasis in case 4–8).

The mutation number of eight CSCC pairs is shown in Figure 1A. Interestingly, the LNM harbored fewer mutations than primary CSCC specimens, although the difference was insignificant (*p* = 0.08, Wilcoxon test). The LM specimens also had more mutations than the LNM specimens without significant difference (*p* = 0.07, Wilcoxon test). In the Venn diagram for the eight cases (Figure 1B), the paired primary lesions and LM shared 28%–70% of mutated genes, while the paired primary lesions and LNM shared 6–47% of mutated genes.

**FIGURE 2:**
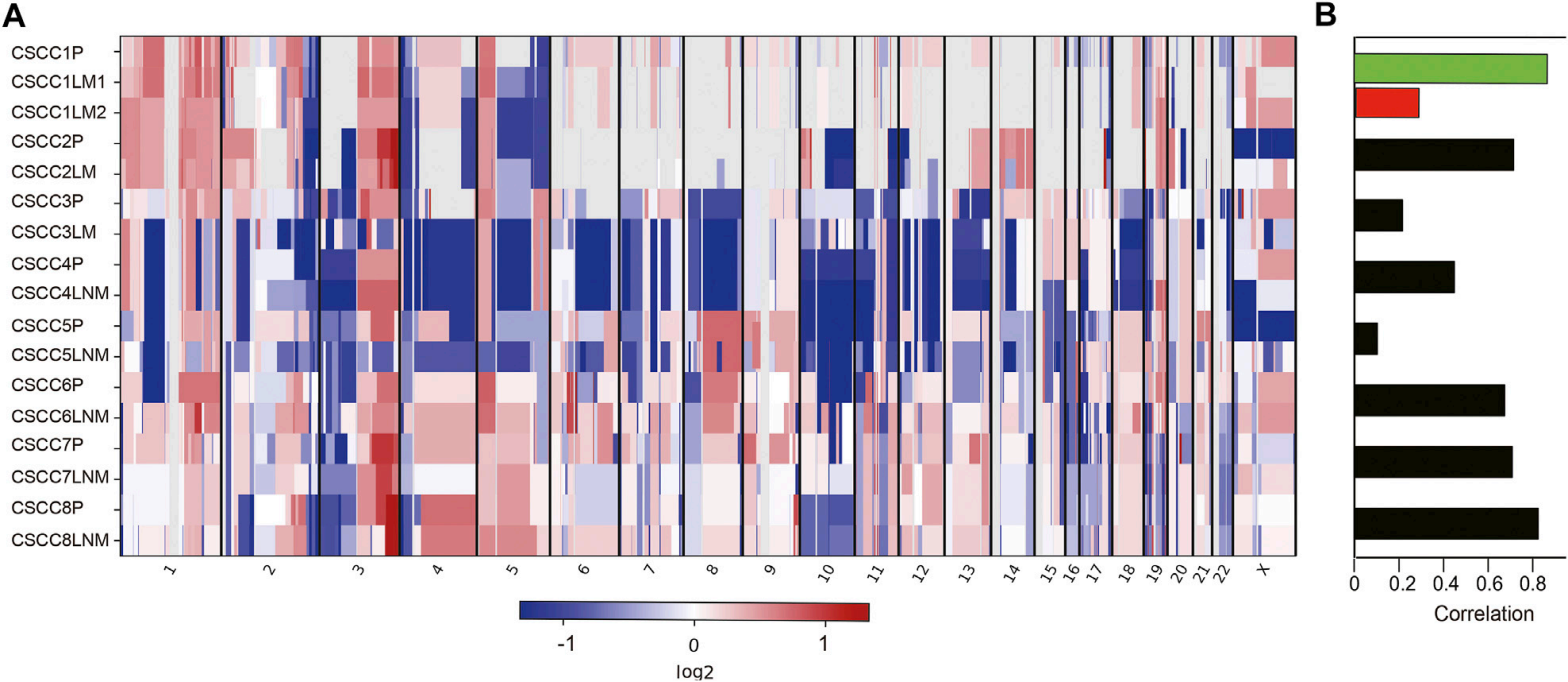
Somatic DNA copy number alterations in each CSCC patient. **(A)** Eight tumor samples were ordered from top to bottom in the chromosome plots, the Log_2_ ratios are plotted on the *y*-axis, and the genomic positions are plotted on the *x*-axis. **(B)** Spearman correlation between primary and matched metastatic CSCC. CSCC: cervical squamous cell carcinoma; P: primary lesions; LM: lung metastatic lesions; LNM: lymph nodes metastatic lesions.

The chromosomal CNVs are a common phenomenon in CSCC [18, 19]. Genome-wide CNA profiling for eight pairs of primary CSCC and matched metastasis genomes are shown in Figure 2 and Supplementary Table S4. The number of CNVs is listed in Supplementary Table S5. CNVs were found to widely exist in primary and metastatic lesions. There was no statistical difference between the CNV number in LM and primary lesions (*p* = 0.593, Wilcoxon test). Similarly, there was no statistical difference in CNV between LNM lesions and primary lesions either (*p* = 0.225, Wilcoxon test). LM1 and LM2 in case 1 had distinct CNV numbers.

Further analysis showed amplification of 9p *CD274* and *PDCD1LG2* (Programmed Cell Death 1 Ligand 2) were detected in the primary lesions of CSCC5 and CSCC7, and LNM lesion in CSCC6.

### Whole-Exome Sequencing Shows Driver Gene Heterogeneity in Primary and Metastatic Specimens

**FIGURE 3:**
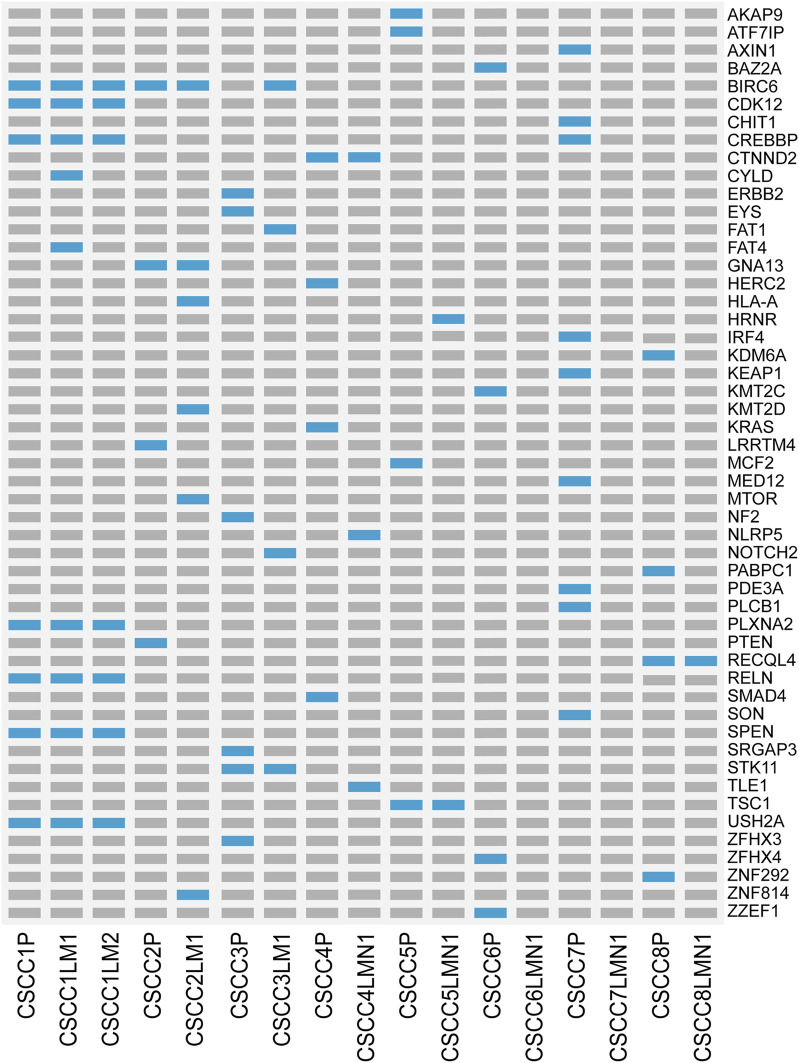
Landscape of somatic driver gene mutations in primary and metastatic CSCC. List of all cancer driver mutations in primary and metastatic CSCC. Light blue bars: single nucleotide variation; grey bars: no mutation was detected. CSCC: cervical squamous cell carcinoma; P: primary lesions; LM: lung metastatic lesions; LNM: lymph nodes metastatic lesions.

Figure 3 and Supplementary Table S3 listed all cancer driver mutations identified in eight patients. The LNM in CSCC6 and CSCC7 did not harbor any driver mutations. The rest of the specimens harbored at least one cancer driver somatic mutation. The driver mutations in LNM were fewer than the primary lesions in all cases (*p* = 0.043, Wilcoxon test). The oncogenic tyrosine kinase pathway was frequently mutated; mutations involving *KRAS* and *PTEN* were detected in the primary specimen of CSCC4 and CSCC2. Gene mutations in the TGFβ signaling pathways could also be found, including *CREBBP* in the primary lesions of CSCC1 and CSCC7, the metastatic lesion in CSCC1.

### Whole-Exome Sequencing Shows the Clonal Relationship Between Primary and Metastatic Specimens

**FIGURE 4:**
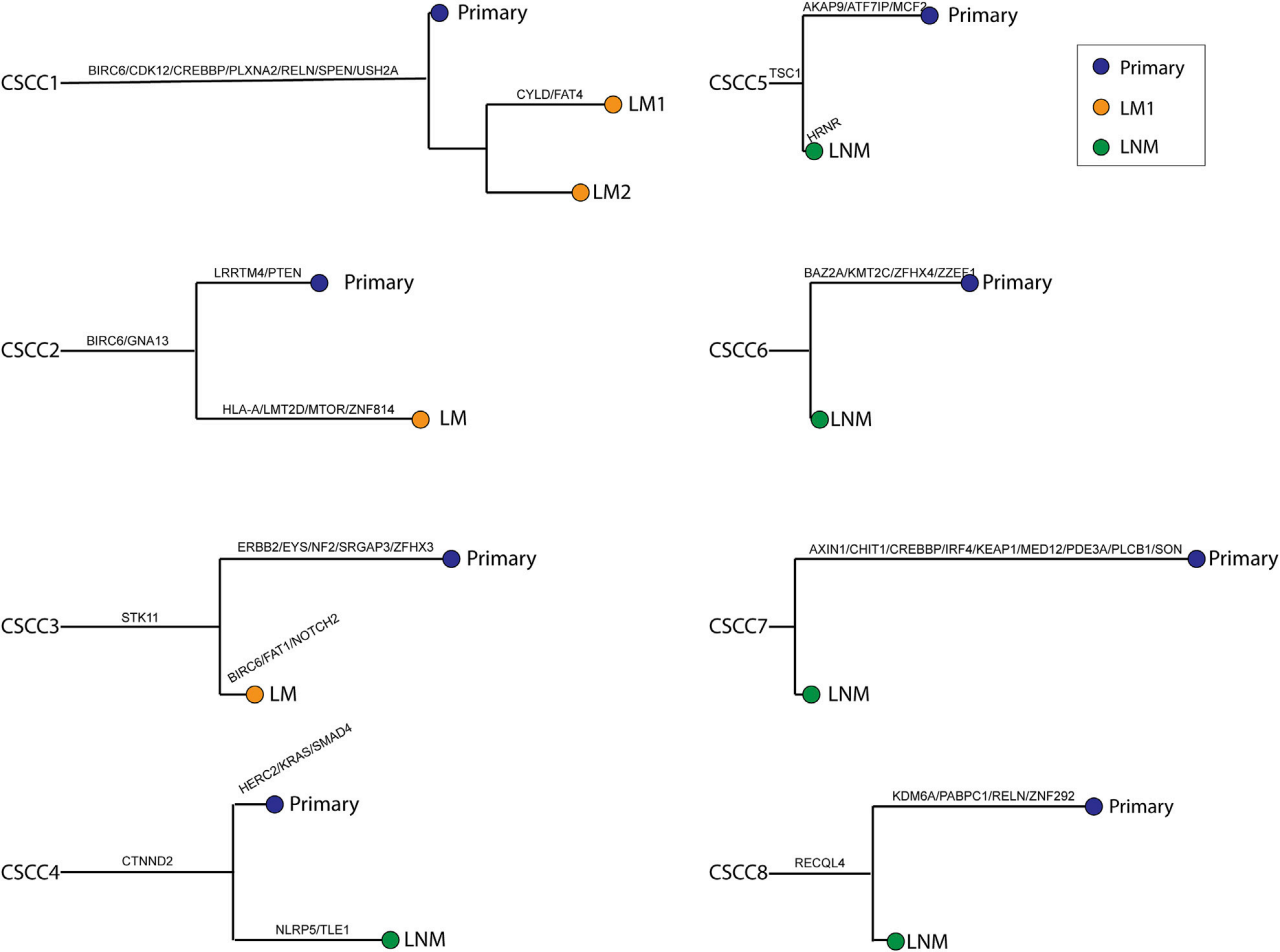
Phylogenetic trees were reconstructed for all cases. Branch length was relative to the number of non-synonymous somatic mutations (not only driver mutations); longer branches indicate more mutations. Branches are labeled with cancer-driver genes. The relative ordering of mutations is for visualization purposes only, as it is impossible to determine which came first. Each leaf corresponds to a single type of tissue harvested from the given patient. CSCC: cervical squamous cell carcinoma; P: primary lesions; LM: lung metastatic lesions; LNM: lymph nodes metastatic lesions.

Figure 4 shows a phylogenetic tree of primary and metastatic lesions. The primary and metastatic lesions shared some mutations, similar to Figure 1B, which meant they were clonally related. And we could distinguish the LM from PPSCC. Further, we tried to develop a next-generation sequencing panel to help distinguish primary and metastatic pulmonary SCC. However, the genes were widely distributed. So, it was hard to apply a gene panel to distinguish the primary and metastatic pulmonary SCC.

## Discussion

This study provides the first comprehensive analysis of the molecular genetic alterations in primary CSCC, LM, and LNM. Despite TCGA publishing the genetic landscape of cervical carcinoma, no genetic study on the metastatic CSCC has been published until now. With the deep sequencing of CSCC, we first established the tumor mutation burden and CNV difference on primary and metastatic CSCC.

The LM is originated from CSCC, which means they shared the original clone. The clones have some mutations, including driver mutations and passenger mutations, so the LM and CSCC shared some mutations. If we could find out the original mutations, or the mutations they shared, we could prove they are clonally related or have the same origin. First, we tried to develop a gene panel to include the common 100 genes of cervical carcinoma from NCBI and hope it could cover the >95% of cervical carcinoma cases, LM and LNM. Then we applied the panel to our data, but it could only distinguish six cases, so we expanded the panel to 500 genes, still failing to distinguish all the metastasis lesions. The first reason is that cervical carcinoma is a virus-driven cancer and the tumor mutations burden is low [20], which means their mutation quantity is low, therefore their shared mutations are pretty limited. The second reason is CSCC does not have specific mutations; the gene mutations are randomly distributed in different cases, therefore a panel could not cover all the cases. Thus, we concluded that WES but not an NGS (next-generation sequencing) gene panel is needed for distinguishing LM and PPSCC.

It is well-known that the accumulation of driver mutations [21] is needed for tumorigenesis, and at least three driver mutations are required for the tumorigenesis of pulmonary and colorectal cancers. Different from this, in this study, the LNM harbored fewer driver mutations than primary specimens and did not harbor any driver mutations in some cases. Thus, the metastasis clone expansion could be a distinct process compared to the tumor progression in primary lesions, it may not need as many driver mutations as the primary lesions. Meanwhile, because the driver mutations and somatic mutations in LNM were fewer than the primary CSCC, the tumor cells may migrate to lymph nodes in a very early stage. After the migration, the tumor cells in primary lesions and metastatic lesions have undergone separate clone development to harbor their private mutation as the phylogenetic tree showed (Figure 4). But the clone development in LNM may be slower than primary lesions because its mutations were fewer than the primary CSCC. Because of the early metastasis of LNM, we need to pay more attention to preventing LNM of early-stage CSCC.

Unlike LNM, LM harbored a similar number of driver mutations with primary lesions, so they may require sufficient driver mutations to expand clones. And the diagnosis for LM and primary CSCC has a time difference of 37 months on average, which means LM have a longer time to accumulate mutations than the primary lesions, but they harbored similar somatic and driver mutation number with primary CSCC so that the clone development may grow slower than the primary lesion because of the different microenvironment. But we do not rule out the possibility that the result is caused by the technical limitations and the involvement of DNA methylation.

From a genetics perspective, there must be mutations that convert primary cancer to a metastatic one, just as there are mutations that convert a normal cell to dysplasia or a carcinoma *in situ* to invasion. However, despite the intensive effort, consistent genetic alterations (including mutations and CNVs) that distinguish metastatic cancers from primary CSCC remain to be identified.

In a large study in 2017 [13], over 70% of cervical cancers exhibited genomic alterations in either one or both of PI3K–MAPK and TGFβ signaling pathways [13]. Like that result, mutations in PI3K–MAPK pathway and TGFβ signaling were detected in a significant percentage of patients in this study while the distribution of these mutations is not specific and could be found in both primary and metastatic lesions. But because of the pathway’s importance, it still needs in-depth research to clarify the role of PI3K–MAPK and TGFβ pathway in the process of oncogenesis in a more significant number of tumors.

One limitation of this study is the limited case number; a detailed sequence study should be performed on a larger cohort in the future. WES could detect SNVs and small indels, but could not recognize large indels, gene fusions, epigenetic changes, or post-translational, transcriptional dysregulations; this is another limitation of our study. Nonetheless, our results shed new light on the pathogenesis of tumor metastasis of CSCC, and our findings have implications for its early detection and prevention.

## Key Messages

(1) Whole-exome sequencing is a helpful tool in distinguishing primary pulmonary squamous cell carcinoma and metastatic cervical squamous cell carcinoma (CSCC).
(2) The lymph nodes metastasis harbored fewer cancer driver mutations than primary CSCC specimens with a significant difference.
(3) There was no significant difference in somatic mutations and copy number variation (CNV) between primary and metastatic CSCC.

## Data Availability

The original contributions presented in the study are included in the article/Supplementary Material, further inquiries can be directed to the corresponding authors.

## Supplementary Material

Supplementary Figure S1 Photomicrographs from a representative case (case 2).
