# Supplementary figures and images for: Whole-Exome Sequencing Could Distinguish Primary Pulmonary Squamous Cell Carcinoma From Lung Metastases in Individuals With Cervical Squamous Cell Carcinoma
Source: Pathol Oncol Res. 2022 May 11;28:1610325. doi: 10.3389/pore.2022.1610325 (PMC9130473; doi:10.3389/pore.2022.1610325)

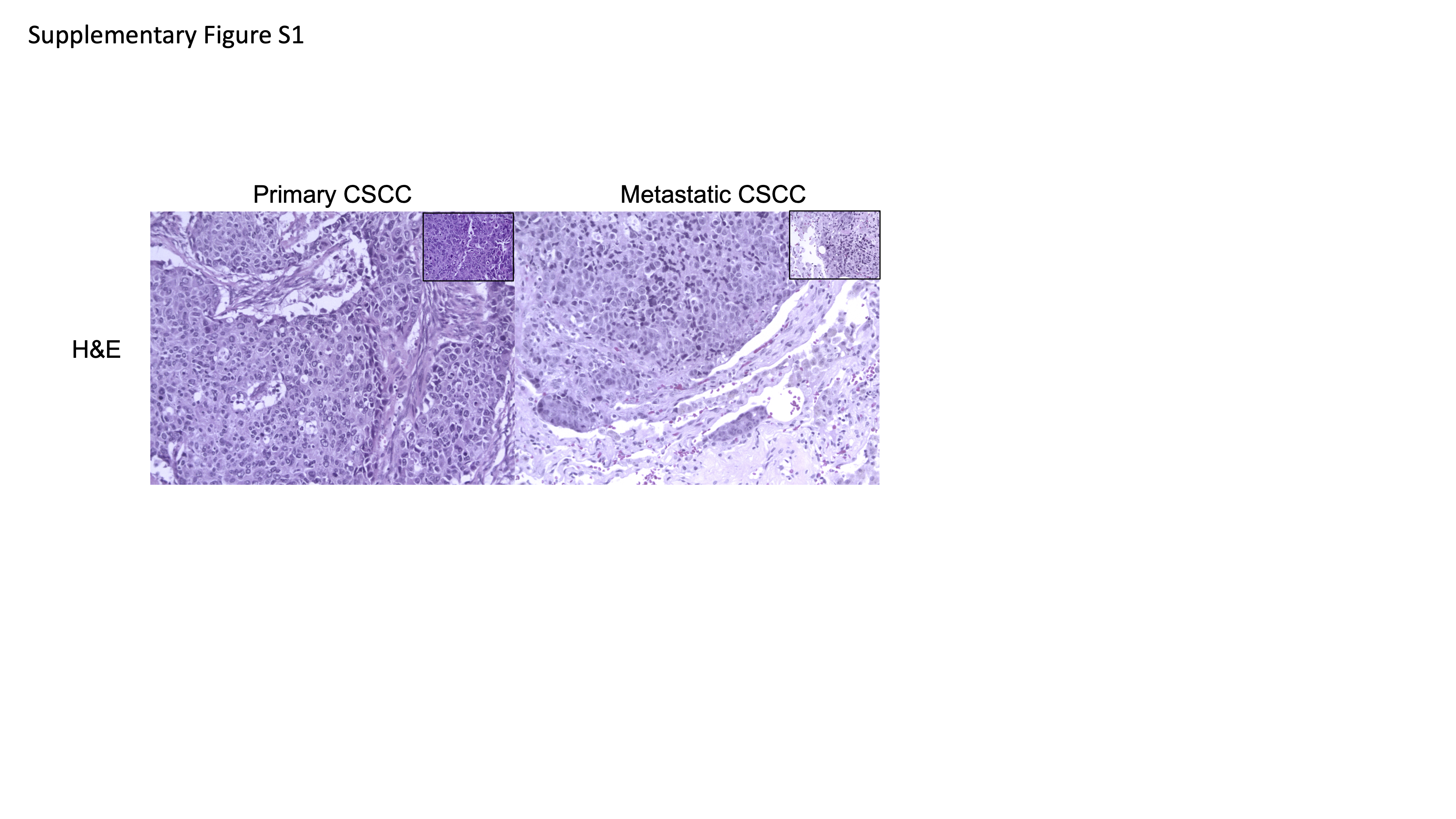

Supplement: Supplementary file 1 [file Image1.JPEG]
